# FGF21 via mitochondrial lipid oxidation promotes physiological vascularization in a mouse model of Phase I ROP

**DOI:** 10.1007/s10456-023-09872-x

**Published:** 2023-03-21

**Authors:** Zhongjie Fu, Pia Lundgren, Aldina Pivodic, Hitomi Yagi, Jarrod C. Harman, Jay Yang, Minji Ko, Katherine Neilsen, Saswata Talukdar, Ann Hellström, Lois E. H. Smith

**Affiliations:** 1 Department of Ophthalmology, Boston Children’s Hospital, Harvard Medical School, Boston, MA 02115 USA; 2 The Sahlgrenska Centre for Pediatric Ophthalmology Research, Department of Clinical Neuroscience, Institute of Neuroscience and Physiology, Sahlgrenska Academy, University of Gothenburg, Gothenburg, Sweden; 3 Department of Ophthalmology, Keio University School of Medicine, Tokyo, Japan; 4 Merck & Co., Inc, South San Francisco, California USA

**Keywords:** FGF21, Adiponectin, Retinopathy of prematurity, Fatty acid oxidation, Hyperglycemia, Retinal vessel

## Abstract

Hyperglycemia in early postnatal life of preterm infants with incompletely vascularized retinas is associated with increased risk of potentially blinding neovascular retinopathy of prematurity (ROP). Neovascular ROP (Phase II ROP) is a compensatory but ultimately pathological response to the suppression of physiological postnatal retinal vascular development (Phase I ROP). Hyperglycemia in neonatal mice which suppresses physiological retinal vascular growth is associated with decreased expression of systemic and retinal fibroblast growth factor 21 (FGF21). FGF21 administration promoted and FGF21 deficiency suppressed the physiological retinal vessel growth. FGF21 increased serum adiponectin (APN) levels and loss of APN abolished FGF21 promotion of physiological retinal vascular development. Blocking mitochondrial fatty acid oxidation also abolished FGF21 protection against delayed physiological retinal vessel growth. Clinically, preterm infants developing severe neovascular ROP (versus non-severe ROP) had a lower total lipid intake with more parenteral and less enteral during the first 4 weeks of life. Our data suggest that increasing FGF21 levels in the presence of adequate enteral lipids may help prevent Phase I retinopathy (and therefore prevent neovascular disease).

**Supplementary Information:**

The online version contains supplementary material available at 10.1007/s10456-023-09872-x.

## Introduction

**Fig. 1:**
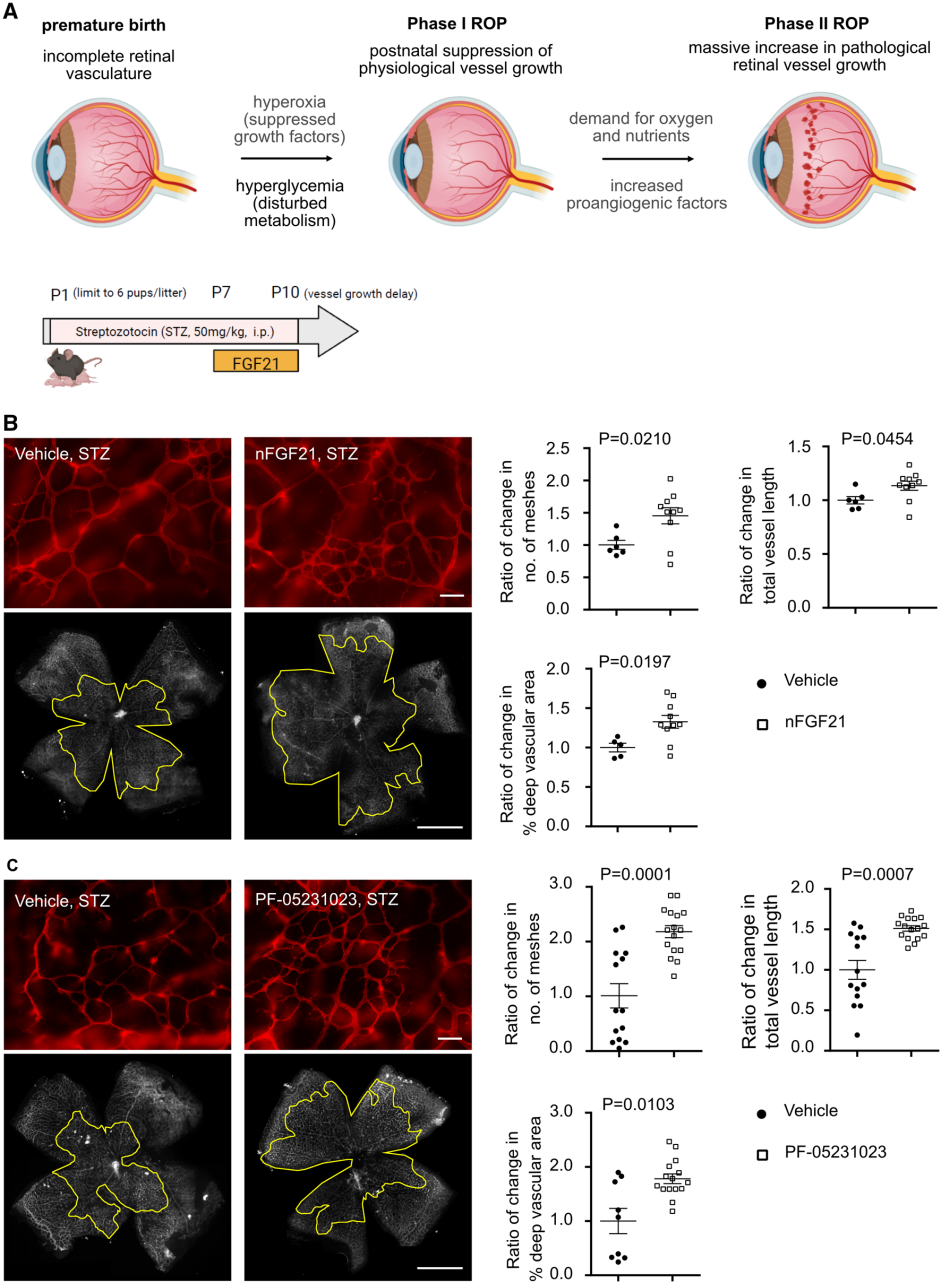
FGF21 administration promoted physiological retinal vascular growth in Phase I ROP. **A** Schematics of ROP development and mouse HAR model. The schematics were generated using BioRender. Native FGF21 (1 mg/kg) or PF-05231023 (0.5 mg/kg) from P7 to P9 was intraperitoneally (i.p.) injected in C57BL/6 J (WT) mouse pups with hyperglycemia-induced suppression of physiological vascular development modeling Phase I ROP. At P10, whole mounted retinas were stained with isolectin (red) and retinal vascular network was quantified. Littermate mice were injected with vehicle as controls. Both native FGF21 (*n* = 5–10 retinas per group, B) and PF-05231023 (*n* = 9–15 retinas per group, C) promoted retinal vessel growth. The outlined area (in yellow) represents the vascular area in the deep plexus. Scale bar: 50 µm (top in B, C), 1 mm (bottom in B, C). Normality (quantile–quantile plot) and *F*-test were first conducted and unpaired *t*-test (**B**) or Welch’s *t*-test (**C**) was used to compare the groups

The incidence of potentially blinding neovascular retinopathy of prematurity (ROP) is increasing with the increased survival of immature preterm infants [1]. Infants born preterm have incompletely vascularized retinas. ROP is initiated at birth with the onset of suppression of the physiological growth of retinal vessels that would have occurred in utero leaving the peripheral retina avascular (Phase I, Fig. 1A). Non-vascularized but maturing retina with increasing energy demand becomes hypoxic and energy deficient which causes the release of vaso-proliferative factors that drive pathological neovascularization (Phase II). Current treatments, including laser therapy and anti-vascular endothelial growth factor (VEGF) therapy [2, 3], focus on suppressing neovascularization (Phase II ROP). Anti-VEGF treatment in excess can also (counter-productively) suppress physiological vascular growth. Early promotion of physiological retinal vessel growth as occurs in utero will prevent Phase I (suppression of physiological vascular growth) which will in turn protect against Phase II (compensatory but pathological vascular growth) and prevent persistent retinal neuronal dysfunction that occurs even after the vascular issue is resolved [4]. Mechanisms behind delayed physiological retinal vascularization are not fully understood. It is well established that hyperoxia causes suppression of physiological retinal vascular growth [5]. However, there is another very important factor contributing to suppression of physiological retinal vascular growth. Perinatal hyperglycemia in the first few weeks of life is a major independent risk factor for both Phase I and Phase II ROP, and the risk increases with the severity and duration of hyperglycemia [6–14]. The mechanisms are not fully known.

We have previously reported that the degree of hyperglycemia in the first two weeks correlates with low white adipose-derived adiponectin (APN) levels and with delayed physiological retinal vascularization in Phase I retinopathy [15]. During fetal development, serum APN levels increase markedly. Low APN levels correlate with low birth weight (BW) and BW standard deviation score (SDS) [16]. After birth, increasing APN levels may help with rapid metabolic adaptation to extrauterine life during the first weeks of life [17, 18]. In preterm infants, APN levels increase rapidly during the first 3 weeks of life, associated with energy intake [19]. The magnitude of an early increase in circulating APN levels in preterm infants is positively associated with later postnatal growth [18–21]. Infants born small for gestational age (SGA) have lower levels of circulating APN at birth and poorer postnatal growth than infants born appropriate for gestational age (AGA) [18–22]. In the mouse model of hyperglycemia-associated Phase I retinopathy, we found that induction of the APN pathway increases photoreceptor metabolism and improves retinal neurovascular development [15]. Loss of APN causes imbalance of essential retinal *ω*-3 and *ω*-6 long-chain polyunsaturated fatty acids, which are major lipid components of photoreceptors [23]. APN is an important metabolic regulator and disrupted APN signaling contributes to several retinal metabolic disorders [24]. However, therapeutic use of recombinant APN is technically challenging given its complex secondary structure which makes it difficult to synthesize. Therefore, we used a known activator of APN, fibroblast growth factor 21 (FGF21), which is a key regulator of APN production and secretion in diabetic patients and mice with hyperglycemia [25, 26].

The role of FGF21 in premature infants and ROP is not well understood. In full-term infants, FGF21, synthesized in the liver [27], is induced from postnatal day 2 through the first year [28]. However, in preterm infants, circulating levels of FGF21 are below the lower limit of quantitation (LLOQ) of the assay in 85% of preterm infants at birth (cord blood), and in 31%, and 25% at 1 and 3 weeks, respectively [29–31]. Interestingly, FGF21 levels in the first 24 h positively correlate with infant weight and length Z-scores at discharge [31]. In the offspring of pregnant women with normal glucose tolerance, cord blood FGF21 levels also positively correlate with postnatal growth of infants [32]. Most preterm infants have poor postnatal growth in part due to inadequate nutrient intake. Although prolonged starvation in humans increases circulating FGF21 levels [33], it does not rise to normal levels in preterm infants with inadequate nutrition [30]. Poor postnatal weight gain predicts neovascular ROP incidence and severity (likely due to poor physiological retinal vascular development [Phase I ROP)] [34, 35]. These findings suggest that deficiency of FGF21 may contribute to neovascular ROP. FGF21 protects against several neurovascular retinal disorders including proliferative ROP, retinitis pigmentosa, diabetic retinopathy, retinal vascular leakage, and neovascular age-related macular degeneration [36–39]. Using FGF21 supplementation and knockout of FGF21, we evaluated if FGF21 promoted physiological retinal vessel growth through activation of the APN pathway in a mouse model of hyperglycemia-induced vascular growth suppression (a model of the contribution of metabolism to Phase I retinopathy). We also tested if FGF21 promotion of physiological retinal vessel growth persisted in APN-deficient mice. We compared our preclinical results in mice with those in a cohort of preterm infants developing severe vs. non-severe neovascular ROP. We examined lipid nutritional intake during their first weeks of life corresponding to the timing of Phase I ROP.

## Methods

### Study approval

All animal studies adhered to the Association for Research in Vision and Ophthalmology Statement for the Use of Animals in Ophthalmic and Vision Research and were approved by the Institutional Animal Care and Use Committee at Boston Children’s Hospital (00,001,619). The Regional Ethical Board in Gothenburg (Dnr 303–11) approved the clinical study.

### Neonatal mouse model of hyperglycemia-associated phase I retinopathy

Daily intraperitoneal (i.p.) injection of streptozotocin (STZ, 50 mg/kg) from P1 to P9 (Fig. 1A) was given to induce hyperglycemia which was first detected around P8 [15], which is when the formation of the superficial vascular layer is mostly complete, but the formation of the next layer, the deep vascular network, is at an early stage. Physiological retinal vascularization of the deep retinal vascular plexus measured at P10 was delayed by hyperglycemia as previously described [15]. No significant changes were found in the superficial retinal vascular plexus at P10. There is no direct toxicity of intravitreal STZ on retinal vessel growth and insulin treatment restores physiological retinal vascularization [15]. The body weight of STZ-treated and non-treated littermate controls was equal. Litter size was limited to a maximum of six pups in the STZ-treated group and eight to nine pups per litter in normoglycemic control groups to achieve comparable body weights [15]. All interventional drugs were administered from P7 to P9 to cover the induction period of hyperglycemia and the early phase of deep retinal vessel growth.

For FGF21 treatment, both native FGF21 (1 mg/kg) and long-acting FGF21 (PF-05231023, 0.5 mg/kg) were administered i.p. daily from P7 to P9 in C57BL/6 J (WT, Jackson Laboratory, #000,664) or APN knockout (*Adipoq*^*−/−*^, Jackson Laboratory, #008,195) mice. Littermate mice were injected with vehicle control (phosphate-buffered saline, PBS). To determine if lipid metabolism mediated FGF21 protection against hyperglycemia-induced suppression of physiological retinal vascular growth (Phase I retinopathy), PF-05231023 (0.5 mg/kg)-treated C57BL/6 J (WT) mice were co-treated with the CPT1A inhibitor etomoxir (4 mg/kg, Sigma, E1905) or vehicle (DMSO) i.p. from P7 to P9. At P10, retinas were whole mounted and stained with isolectin GS-IB_4_ (vessel marker, Invitrogen, I21413). The retinal deep vascular network formation (number of meshes, total vessel length) was evaluated using “*Angiogenesis Analyzer*” plugin in Image J as previously reported [15]. Both female and male pups were used. Body weight and blood glucose levels were recorded. Serum triglycerides levels were quantified using the Wako *L*-Type TG M test.

FGF21 knockout (KO) (*Fgf21*^*−/−*^) and WT (*Fgf21*^+*/*+^) mice were kindly provided by Dr. Steven Kliewer, University of Texas Southwestern Medical Center. We first generated heterozygous FGF21 mice as breeding stock. Postnatal hyperglycemia was induced with STZ and the retinal vasculature of *Fgf21*^*−/−*^ and littermate WT *Fgf21*^+*/*+^ mice was compared in P10 whole mounted retinas.

### Western blot

To preserve the secondary structure of APN, serum (1 µl) from FGF21 (PF-05231023) vs. vehicle-treated Phase I retinopathy mice was incubated with Laemmli’s SDS sample buffer (BP-110R; Boston BioProducts Inc.) for 1 h at room temperature [40]. Primary antibody against APN (1:1000, AF1119; R&D) was used. Signals were visualized with 1:5000 corresponding horse-radish peroxidase-conjugated secondary antibodies and enhanced chemiluminescence (Pierce). The band signal intensity was quantified using Image J. Seven mice per group were used.

Two retinas from the same mouse were pooled for protein extraction using RIPA lysis buffer (R0278, Sigma-Aldrich) with protease inhibitor (1:1000, P-8340, Sigma-Aldrich) and phosphatase inhibitor (1:100, P0044, Sigma-Aldrich). Primary antibodies targeting phospho-TFEB (Ser142) (1:1000, ABE 1971, EMD Millipore), TFEB (1:2000, A303-673A, Bethyl Laboratories) were used. Signals were visualized with 1:5000 corresponding horse-radish peroxidase-conjugated secondary antibodies and enhanced chemiluminescence (Pierce). β-ACTIN (1:2500, A1978, Sigma-Aldrich) was used as internal control. The signal of band intensity was quantified using Image J. The levels of target proteins were first referred to the levels of β-ACTIN. Ratio of change was then calculated referring to the vehicle control group as one. Six mice per group were used.

### Immunohistochemistry (IHC)

IHC was conducted as previously reported [39, 41]. P10 C57BL/6 J mouse retinal cryosections were first treated with ice-cold methanol and rinsed with 0.1% triton PBS. The sections were then incubated with 2% bovine serum albumin for 1 h at room temperature and stained with primary antibody against FGFR1 (1:200, ab58516, Abcam) overnight at 4 °C. The sections were washed with PBS and then stained with a corresponding secondary antibody, covered in mounting medium with 4′,6-Diamidine-2′-phenylindole dihydrochloride (DAPI, H-1200, Vector laboratories) for cell nucleus. The immunosignals were visualized with a Zeiss confocal microscope at 200X magnification.

### Clinical data

This study analyzed longitudinal blood samples of 14 infants born GA < 28 weeks at Sahlgrenska University Hospital in Gothenburg, Sweden, from April 2013 to September 2015. The infants had been included in the Donna Mega study (Clinical trial NCT 02,760,472). The Regional Ethical Board in Gothenburg (Dnr 303–11) approved the study. The mean GA for preterm infants with non-severe and severe neovascular ROP was 26.8 (SD ± 1.0) vs 24.2 (SD ± 1.0) (*P* = 0.004), mean birthweight was 984.3 (SD ± 218.9) vs. 592.1 (SD ± 136.6) (*P* = 0.006), and mean BW standard deviation score was − 0.54 (SD ± 0.8) vs. − 1.3 (SD ± 1.9) (*P* = 0.949). Blood samples were taken on postnatal days (PNA) 1, 7, 14, and 28, and at postmenstrual age (PMA) at 32 weeks according to study protocol [42]. Nutritional intake, total, enteral and parenteral energy (kcal/kg/day), and lipids (g/kg/day) were presented as mean values for the following time periods: first PNA week (PNA days 2–7), second PNA week (PNA days 8–14), and PNA weeks 3 and 4 (PNA days 15–28), details previously presented [42]. International guidelines for ROP classification and ROP treatment were followed [43, 44]. ROP was categorized as “non-severe ROP” (no ROP or ROP stages 1 and 2) and “severe ROP” (ROP stage 3 or more, treated or not treated).

### Statistics

For experimental studies, data are presented as Mean ± SEM. Normality (quantile–quantile plot). F-test (for variance) was first conducted, and parametric unpaired *t*-test (or Welch’s *t*-test) and non-parametric Mann–Whitney test were used to compare the groups (Prism v9.0; GraphPad Software, Inc., San Diego, CA). *P* < 0.05 was considered as statistically significant. For clinical studies, normality (quantile–quantile plot) and *F*-test (for variance) were first conducted, and parametric two-sample *t*-test (or Welch’s *t*-test) or non-parametric Mann–Whitney test was used to compare the severe vs non-severe ROP groups, based on the visual review of the diagnostic plots. Statistical significance was conducted at the level of 0.05. Analyses were performed using SAS software version 9.4 (SAS Institute Inc., Cary, NC, US). Due to the small sample size (*n* = 14) no adjusted analyses were performed.

## Results

### FGF21 administration promoted physiological retinal vascular growth in phase I retinopathy

In hyperglycemic mouse pups with suppression of physiological vascular development (modeling Phase I retinopathy), there were decreased retinal and liver mRNA expressions of *Fgf21* (Supplementary Fig. 1), similar to failed FGF21 induction in preterm infants immediately after birth (who are commonly hyperglycemic). To investigate if FGF21 administration protects against hyperglycemia-associated retinal vessel growth delay, native FGF21 or long-acting FGF21 (PF-05231023) was injected i.p. from P7 to P9 in STZ-induced hyperglycemic C57BL/6 J (WT) mouse pups. At P10, both native FGF21 (1 mg/kg, Fig. 1B) and long-acting FGF21 analogue PF-05231023 (0.5 mg/kg, Fig. 1C) promoted retinal vessel growth compared to littermate vehicle controls. Body weight and blood glucose levels were comparable between FGF21 vs. vehicle groups with averages around 4 g and 140 to 160 mg/dl, respectively. In runty mice (2.0–3.5 g), native FGF21 (1 mg/kg) not only strongly promoted retinal vessel growth but also promoted body weight gain (Supplementary Fig. 2), suggesting that FGF21 is an important growth factor particularly in those with poor postnatal weight gain.

### FGF21 deficiency worsened physiological retinal vascular development in phase I retinopathy

**Fig. 2:**
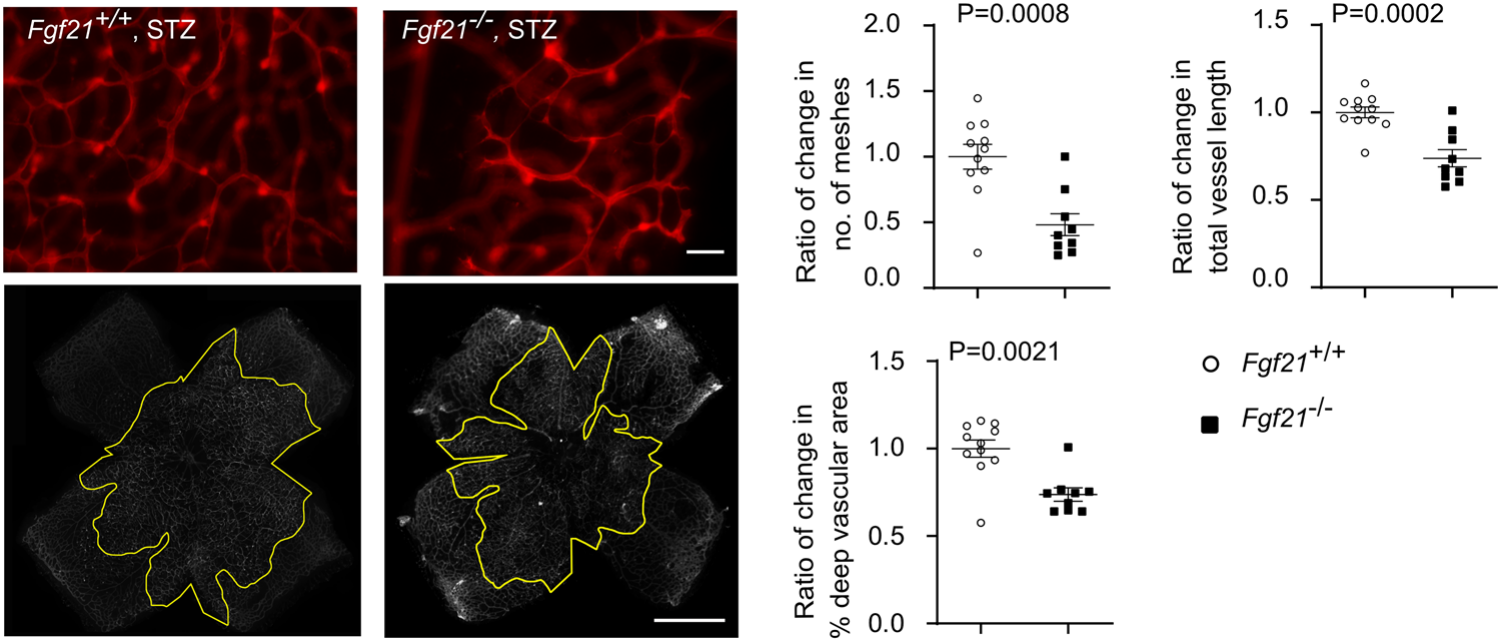
FGF21 deficiency further delayed retinal vascular development in hyperglycemia-induced suppression of physiological vascular development modeling Phase I ROP. Decreased retinal vascular network parameters measuring vascular development at P10 were found in *Fgf21*^*−/−*^ (knockout) vs. littermate *Fgf21* ^+/+^ (WT) mouse pups with hyperglycemia-induced suppression of physiological vascular development modeling Phase I ROP. The outlined area (in yellow) represents the vascular area in the deep plexus. Scale bar: 50 µm (top), 1 mm (bottom). *n* = 9–11 retinas per group. Normality (quantile–quantile plot) and *F*-test were first conducted, and unpaired *t*-test was used to compare the groups

Retinal vessel growth was compared in *Fgf21*^*−/−*^ mice and littermate *Fgf21*^+*/*+^ (WT) mice at P10 during both normoglycemic and hyperglycemic conditions. Loss of FGF21 further attenuated physiological retinal vessel growth in hyperglycemia-associated vascular growth suppression (Phase I retinopathy) (Fig. 2) and also attenuated physiological retinal vessel growth under normoglycemic conditions (Supplementary Fig. 3). Body weight and blood glucose levels were comparable between hyperglycemic *Fgf21*^+*/*+^ and *Fgf21*^*−/−*^ mice with means of 4.3 to 4.6 g and 131 to 154 mg/dl, respectively. These findings further suggest that FGF21 is a key regulator of physiological retinal vascularization during development.

### Adiponectin (APN) mediated FGF21 promotion of physiological retinal vascular development

Low circulating APN levels are associated with hyperglycemia and delayed physiological retinal vascularization in preterm infants [15]. In hyperglycemia-associated physiological vascular growth suppression (Phase I retinopathy) mice, we have demonstrated that activation of the APN pathway improves physiological retinal vessel growth [15, 23]. FGF21 increases APN secretion in diabetic (hyperglycemic) patients and mice [25, 26]. We have previously shown that FGF21 also promotes physiological retinal re-vascularization and inhibits pathological retinal neovascularization in mouse oxygen-induced retinopathy (modeling neovascularization of Phase II ROP) in large part, via white adipose tissue-derived APN [36].

**Fig. 3:**
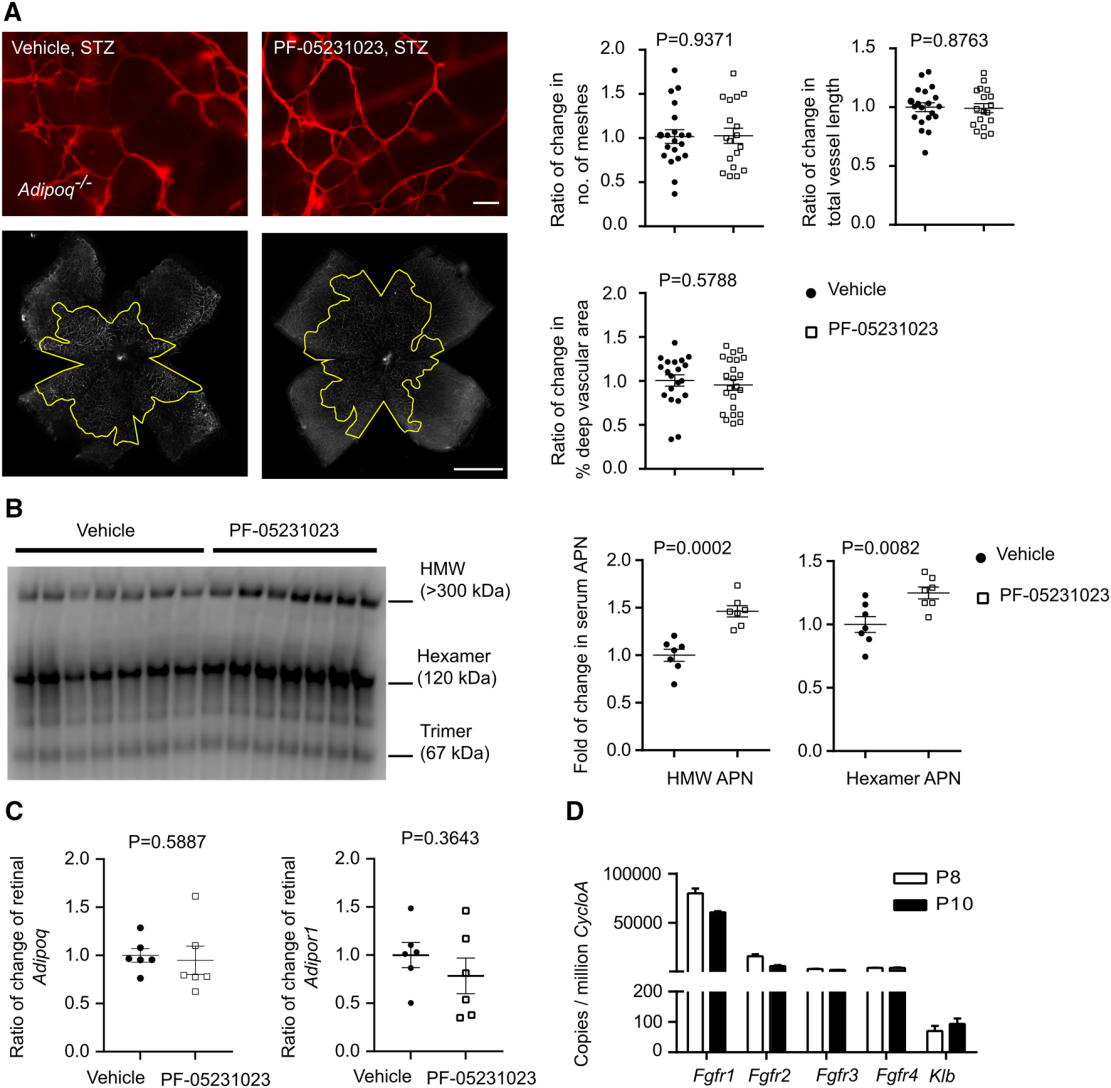
FGF21 mediated through adiponectin (APN) promoted retinal vascular development in a model of Phase I ROP. **A** FGF21 (PF-05231023, 0.5 mg/kg, i.p., P7–9) promotion of retinal vessel growth was lost in Adipoq−/− (KO) mice with hyperglycemia-induced suppression of physiological vascular development modeling Phase I ROP. Adipoq−/− mice were treated with PF-05231023 (0.5 mg/kg, ip, P7–9) or vehicle. The outlined area (in yellow) represents the vascular area in the deep plexus. Scale bar: 50 µm (top), 1 mm (bottom). *n* = 18–23 retinas per group. **B** FGF21 (PF-05231023, 0.5 mg/kg, i.p., P7–9) vs. vehicle increased serum APN levels in C57BL/6 J (WT) mice with hyperglycemia-induced suppression of physiological vascular development modeling Phase I ROP. 1ug serum from each mouse was used for Western blot. *n* = 7 mice per group. **C** FGF21 (PF-05231023, 0.5 mg/kg, i.p., P7–9) vs. vehicle did not change retinal gene expression of Adipoq and Adipor1 in C57BL/6 J (WT) mice with hyperglycemia-induced suppression of physiological vascular development modeling Phase I ROP. *n* = 6 mice per group. **D** FGF21 receptors and its essential co-receptor (Klb) were present in normal neonatal mouse retinas at P8 and P10. *n* = 3 replicates per time points. Six retinas were pooled as *n* = 1. Normality (quantile–quantile plot) and *F*-test were first conducted and unpaired *t*-test or Mann–Whitney test was used to compare the groups

Here, we examined if FGF21 protects against hyperglycemia-associated delay of physiological retinal vessel growth in WT mice mediated through APN (Fig. 1). Long-acting FGF21 (PF-05231023) or vehicle control (PBS) was injected i.p. from P7 to P9 in STZ-induced hyperglycemic APN KO (*Adipoq*^*−/−*^) mice. In APN KO mice, FGF21 (PF-05231023) no longer promoted physiological retinal vascular development (Fig. 3A). FGF21 (PF-05231023) vs. vehicle control significantly increased circulating APN levels, particularly the high-molecular-weight (HMW, bioactive) and hexamer forms (Fig. 3B) and did not affect retinal mRNA expression of *Adipoq* and APN receptor *Adipor1* (Fig. 3C). Loss of APN did not affect hyperglycemia in FGF21 vs. vehicle-treated Phase I retinopathy mice (blood glucose averaged 150 to 160 mg/dl). These findings suggest that FGF21 promoted physiological retinal vessel growth in Phase I retinopathy mediated through increased circulating APN. There is also a potential direct impact of FGF21 on retinas as FGF21 receptors and co-receptors were expressed in neonatal mouse retinas (Fig. 3D). Immunostaining of FGFR1 showed positive signals at deep retinal vascular plexus at P10 neonatal mouse retina and single-cell analysis also showed gene expression of *Fgfr1* in endothelial cells and Muller glia at P12 neonatal mouse retina (Supplementary Fig. 4).

### FGF21 controlled physiological retinal vessel growth by modulating lipid metabolism

**Fig. 4:**
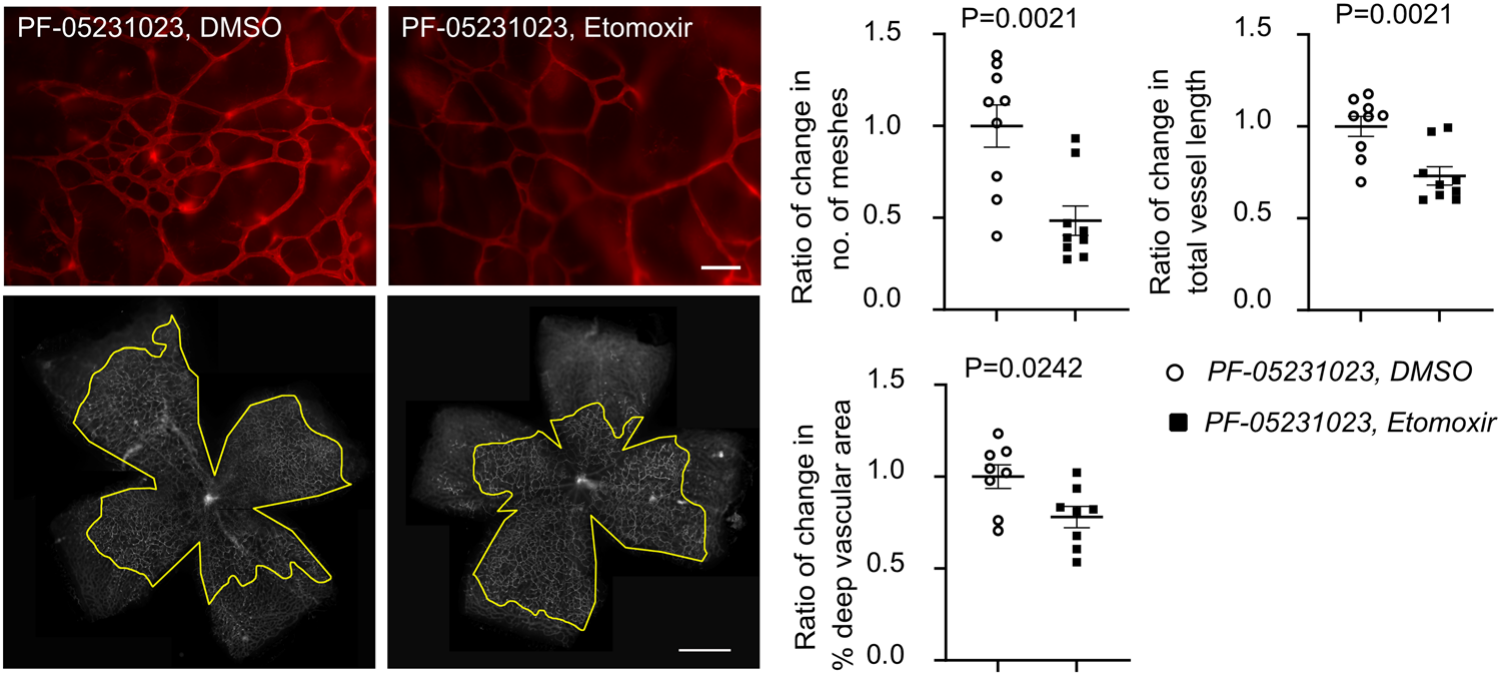
FGF21 required mitochondrial lipid oxidation to improve vessel growth in hyperglycemia-induced suppression of physiological vascular development modeling Phase I ROP. Inhibition of mitochondrial lipid transporter abolished FGF21 promotion of retinal vessel growth. C57BL/6 J (WT) mice were co-treated with PF-05231023 (0.5 mg/kg, ip, P7–9) and etomoxir (4 mg/kg, ip, P7–9) or vehicle (DMSO). The outlined area (in yellow) represents the vascular area in the deep plexus. Scale bar: 50 µm (above), 1 mm (below). *n* = 8–9 retinas per group. Normality (quantile–quantile plot) and *F*-test were first conducted and unpaired *t*-test was used to compare the groups

FGF21 is an essential lipid metabolic modulator. Clinical trials of long-acting FGF21 in type 2 diabetic patients show an improved circulating lipid profile (decreased triglycerides) but little or no impact on glycemic control [26, 45]. In hyperglycemia-associated Phase I retinopathy mice, we also found that FGF21 (PF-05231023) treatment decreased circulating triglyceride levels. However, this effect was lost with APN deficiency (Supplementary Fig. 5). To determine if lipid metabolism is key in FGF21 promotion of retinal vessel growth in Phase I retinopathy mice, FGF21 (PF-05231023, 0.5 mg/kg) was co-injected with etomoxir (4 mg/kg), the inhibitor of mitochondrial lipid transporter CPT1A to block fatty acid oxidation [46], from P7 to P9. Etomoxir largely abolished FGF21 promotion of physiological retinal vessel growth evaluated at P10 (Fig. 4). Taken together, FGF21 modulated the circulating lipid profile (decreased triglycerides) and improved retinal vessel growth through mitochondrial fatty acid beta oxidation. Interestingly, we also found that hyperglycemia increased in neonatal mice co-treated with FGF21 (PF-05231023) and etomoxir (292.4 ± 30.51 mg/dl) vs. FGF21 (PF-05231023) and vehicle (DMSO, 186.6 ± 15.29 mg/dl, *P* = 0.0159).

### FGF21 activated the TFEB pathway to control lipid metabolism

**Fig. 5:**
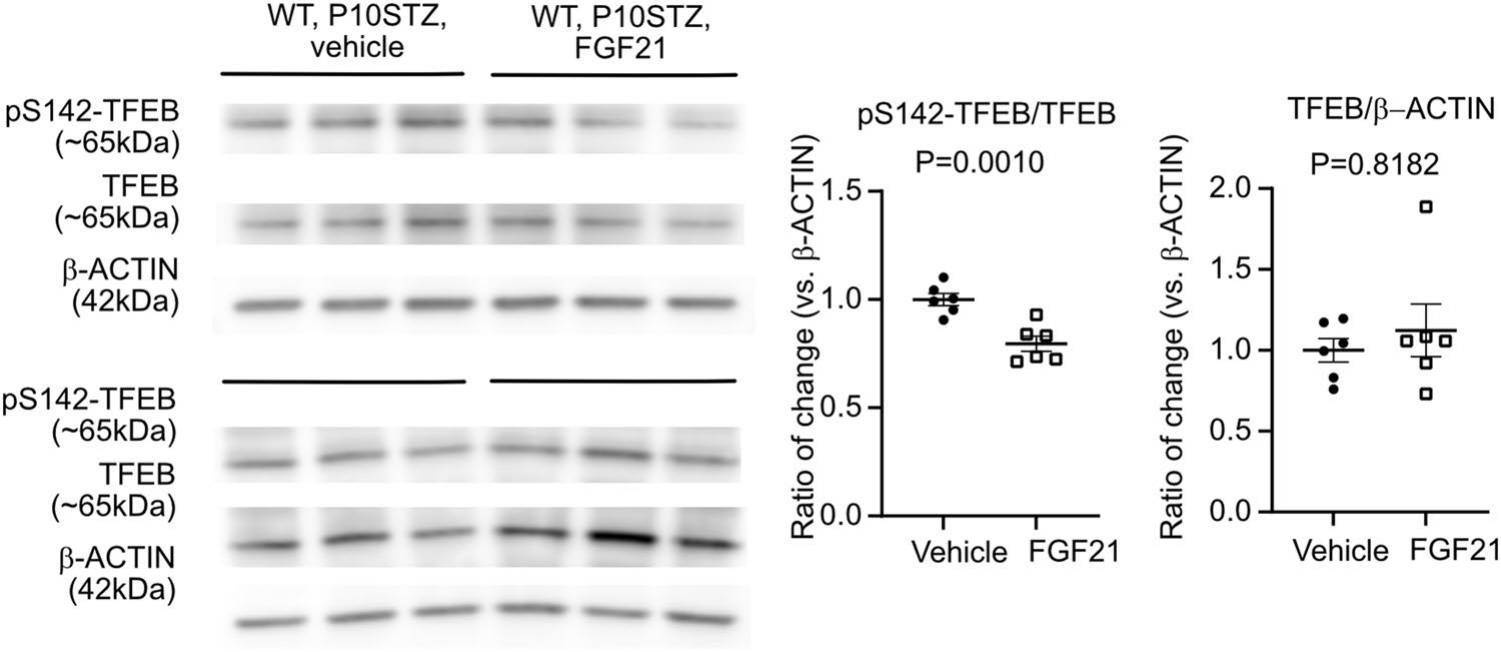
FGF21 activated the TFEB pathway. FGF21 (PF-05231023) vs. vehicle decreased phosphorylation of TFEB at serine 142. *β*-ACTIN served as internal control. Two retinas from the same mouse were pooled as *n* = 1. Western blots were conducted with two independent rounds with *n* = 3 mice per group each round. Normality (quantile–quantile plot) and *F*-test were first conducted, and unpaired *t*-test or Mann–Whitney test was used to compare the groups

We next investigated the potential regulators mediating the actions of FGF21 on lipid metabolism. TFEB is a factor–controlling autophagy and lipid homeostasis [47, 48]. Phosphorylation of TFEB at serine142 blocks TFEB translocation into the nucleus and activation of downstream lipid metabolism [49]. In hyperglycemia-associated Phase I retinopathy mice, FGF21 (PF-05231023) decreased TFEB phosphorylation at serine 142 (Fig. 5), suggesting activation of the TFEB pathway by FGF21.

### Preterm infants with severe ROP had inadequate lipid intake

**Fig. 6:**
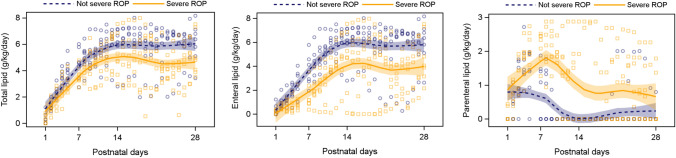
Total, enteral, and parenteral lipid intake (g/kg/day) over first 4 weeks of postnatal age in infants with and without severe ROP. ROP = retinopathy of prematurity

In a clinical study of fourteen infants, seven infants were categorized as having “severe ROP” (all seven had stage 3 ROP and six had received ROP treatment) and seven with “non-severe ROP” (four had no ROP, one had stage 1 ROP, and two had stage 2 ROP). Infants developing severe ROP received less total lipid (and less enteral lipid and more parenteral lipid) during their first 4 weeks of life than infants without severe ROP. Most pronounced differences were observed during the second week of life (enteral lipid intake 5.5 vs 3.4 g/kg/d, *P* = 0.019, in non-severe ROP vs. severe ROP groups, respectively, parenteral lipid intake 0.13 vs 1.44 g/kg/d, *P* = 0.009, respectively (Fig. 6 and Supplementary Table 1).

## Discussion

Oxygen is a major factor suppressing physiological retinal vascular growth in preterm infants and supplemental oxygen is carefully controlled. Although less well studied, abnormal metabolism also has a major impact on suppressing physiological retinal vessel growth and may be amenable to interventions that promote normal vascular growth that will then prevent pathological neovascular ROP. Early perinatal metabolic disturbance (hyperglycemia and dyslipidemia) is associated with ROP progression in preterm infants. We hypothesized that FGF21 may help prevent neovascular ROP by improving physiological retinal vascularization. Preventing Phase I will in turn prevent progression to Phase II of ROP. We found that liver and retinal mRNA expressions of FGF21 were lower in mouse neonates with hyperglycemia-induced suppression of physiological retinal vessel growth. FGF21 administration promoted and deficiency of FGF21 worsened physiological retinal vascularization. The protective effects of FGF21 were mediated by increased APN levels and FGF21 modulated lipid metabolism (mitochondrial fatty acid beta oxidation).

Prior publications suggest that FGF21 is important for glucose and lipid use and body growth in early development [50]. In preterm versus full-term infants, FGF21 levels immediately after birth are markedly reduced [28–30], possibly contributing to poor postnatal growth of infants [32]. Liver FGF21 production and secretion are induced in female mice during late pregnancy [51]. FGF21 is also found in rodent and human breast milk [52] and radiolabeled FGF21 administered to lactating dams accumulates in milk and is transferred to neonatal gut [52], where FGF21 induces gene expression of intestinal peptides and digestive enzymes, controlling intestinal function [52]. We found that in mouse pups with induced insulin deficiency and postnatal hyperglycemia, liver production of FGF21 was decreased, suggesting the potential loss of metabolic regulation in immature liver. Serum triglycerides accumulated in mice with postnatal hyperglycemia as previously reported [15]. We have now shown that FGF21 reduced serum triglycerides without changes in blood glucose levels, suggesting that FGF21 predominantly controls postnatal lipid homeostasis. In mouse pups with extremely low body weight, FGF21 also markedly improved postnatal weight gain, suggesting that FGF21 might be most essential in those infants with more severe metabolic stress. However, FGF21 promoted weight gain in small mouse pups in the setting of adequate nutrition since pups were provided milk from well-fed mothers on demand. In premature infants, an optimal feeding regimen is unknown, but in most cases, nutrition is insufficient for caloric and specific lipid needs.

We have found that in a small cohort of preterm infants with severe ROP versus non-severe ROP, there was less enteral and more parenteral lipid intake, especially in the first two weeks of life. Preterm infants obtained their nutrients mainly through parenteral nutrition during the first days of life and gradually switched to enteral intake of human breast milk as the gut matured. Serum metabolites associated with severe ROP tend to be negatively correlated with enteral nutritional intake in preterm infants [53]. Dietary intake of mother’s milk but not parenteral lipid emulsion correlates with serum docosahexaenoic acid (DHA) and eicosapentaenoic acid (EPA) levels in preterm infants [54], suggesting that it is difficult to increase serum lipids through parenteral lipid supplementation. Breast milk lipidome is associated with early growth in preterm infants [55], possibly due to more medium-chain saturated fatty acid, sphingomyelin, and dihomo-γ-linolenic acid-containing phospho-ethanolamine. Therefore, lipid nutrient shortage was more profound in preterm infants with severe ROP with poor body growth. As mitochondrial fatty acid oxidation is key in mediating FGF21 promotion of physiological retinal vascularization, inadequate lipid status may dampen the effects of FGF21. Further analyses regarding FGF21, and lipid intake would be needed in preterm infants with and without ROP to better understand the relationship.

We investigated a potential regulator, TFEB, influencing FGF21 control of retinal lipid use. In obese mice with FGF21 deficiency, there is impaired control of cardiac autophagy [56]. Starvation induces early TFEB migration from cytoplasm to the nucleus and increases TFEB production in an autoregulatory loop [57]. TFEB regulates genes involved in lipophagy and neutral lipolysis, as well as lipid transport [47]. In mice with disturbed uptake of triglyceride-derived fatty acids and pathological retinal angiogenesis, excess circulating lipids suppress retinal TFEB and decrease sirt-3 and mitochondrial respiration [58]. We found that FGF21 activated the TFEB pathway by decreasing the phosphorylation of TFEB at site ser142, which prevents TFEB entry into the nucleus and localize to the cytoplasm [49]. These observations corresponded to our finding that mitochondrial fatty acid oxidation was a key step to mediate FGF21 effects as inhibiting CPT1A, to block the entry of lipids into the mitochondria and in turn fatty acid oxidation, abolished FGF21 promotion of physiological retinal vessel growth. In preterm infants, higher circulating malonyl carnitine levels (reflecting high malonyl CoA, which also inhibits the mitochondrial lipid transporter CPT1A) correlate with later development of ROP [59]. This suggests that diminished fatty acid oxidation is associated with the development of ROP.

Hyperoxia is also a very strong risk factor suppressing physiological vascular growth in Phase I ROP. We have previously shown that FGF21 promotes normal retinal vessel growth in mice with oxygen-induced retinopathy where hyperoxia suppresses retinal vascular development (Phase I retinopathy) [36]. In oxygen-induced retinopathy, loss of APN also abolishes the FGF21 promotion of physiological retinal re-vascularization [36]. In preterm infants, low APN levels correlate with high perinatal blood glucose levels and delayed retinal vascularization [15]. APN controls photoreceptor metabolism and secretion of growth factors, as well as levels of essential long-chain polyunsaturated fatty acids needed for retinal vessel growth [15, 23]. Increasing APN also helps prevent proliferative Phase II ROP [40]. In addition, APN receptor agonist activates TFEB signaling to inhibit cell proliferation and migration in cultured arterial smooth muscle cells [60]. We therefore concluded that APN is a key mediator for FGF21 to improve the physiological retinal vascularization in early retinopathy. Taken together with our previous report, FGF21 decreased hyperglycemia- and hyperoxia-induced retinal vascular suppression of Phase I retinopathy and reduced hypoxia-induced retinal neovascularization in Phase II. These findings are consistent with the concept that promotion of physiological retinal vascularization improves the nutrient and oxygen supply to the immature neural retina, thus reducing the risk for uncontrolled retinal vessel proliferation driven by nutrient deprivation and hypoxia.

## Conclusion

**Fig. 7:**
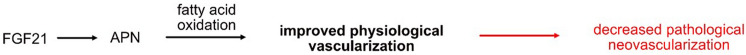
Schematic illustration of proposed pathway for FGF21’s role in hyperglycemia-associated Phase I ROP

Our experimental investigations demonstrated that FGF21 reversed hyperglycemia-induced suppression of physiological retinal vascularization (Phase I retinopathy) by increasing circulating APN and modulating fatty acid oxidation (Fig. 7). This observation corresponds with our clinical finding that during the first month of life, preterm infants have a lower risk of severe ROP with more total and more enteral lipid intake. We speculate that reduced total lipid intake, particularly enteral (from human milk) as opposed to parenteral lipids, caused nutrient shortage in these infants. We did not observe significant changes in blood glucose levels in hyperglycemic mice, suggesting that FGF21 mainly targeted lipid pathways rather than lowering hyperglycemia. This result is in line with clinical trial observations that FGF21 modulates lipid but does not markedly improve glycemic control in type 2 diabetes [26, 45]. In type 2 diabetes, circulating FGF21 levels are biomarkers for diabetic retinopathy and severe diabetic retinopathy [61]. We speculate that FGF21 might control diabetic retinopathy by modulating the lipid profile. Further elucidation of the types of lipids (long chain, short chain, medium chain) modulated by FGF21 is important, because modulation of the lipid supply to prevent ROP (and diabetic retinopathy) is feasible. It is also important to further investigate lipid and glucose metabolic interaction as blocking mitochondrial fatty acid oxidation with etomoxir worsened hyperglycemia in mice. We speculate that decreased lipid uptake may cause lipid accumulation and in turn signal the retina to reduce glucose uptake, in line with our previous report that accumulated extracellular lipids block glucose uptake in mouse retinas [46].

Our current study has limitations: (1) our mouse model only mimics hyperglycemia induced by insulin deficiency but not insulin resistance which is also observed in preterm infants; (2) the underlying mechanisms behind FGF21 regulation of TFEB pathway and lipid metabolism need to be further explored; (3) in the clinical study, the analyses of nutrient intake in preterm infants were not adjusted for GA and BW due to the small number of patients. A larger cohort is needed to further strengthen the data. Analyses of circulating FGF21, APN, and lipid intake in preterm infants are also needed to enhance the translational value of the current findings.

## Supplementary Information

Below is the link to the electronic supplementary material.

Supplementary file1 (PDF 804 KB)

## Data Availability

All the data supporting the conclusions of this study are included within the article and supplementary data. All the other data and materials are available upon request to the corresponding author.
