# Supplementary material for: FGF21 via mitochondrial lipid oxidation promotes physiological vascularization in a mouse model of Phase I ROP
Source: Angiogenesis. 2023 Mar 21;26(3):409–21. doi: 10.1007/s10456-023-09872-x (PMC10328855; doi:10.1007/s10456-023-09872-x)
Supplement: Supplementary file 1 — Supplementary file1 (PDF 804 KB) [file 10456_2023_9872_MOESM1_ESM.pdf]

Supplementary Materials

FGF21 via mitochondrial lipid oxidation promotes physiological vascularization in a mouse model of Phase I ROP

Zhongjie Fu[1], Pia Lundgren[2], Aldina Pivodic[2], Jarrod Harman[1], Jay Yang[1], Minji Ko[1], Hitomi Yagi[1,3], Katherine Neilsen[1], Saswata Talukdar[4], Ann Hellström[2] and Lois E.H. Smith[1,*]

[1]Department of Ophthalmology, Boston Children's Hospital, Harvard Medical School, Boston, MA, USA.

[2]The Sahlgrenska Centre for Pediatric Ophthalmology Research, Department of Clinical Neuroscience, Institute of Neuroscience and Physiology, Sahlgrenska Academy, University of Gothenburg, Gothenburg, Sweden.

[3]Ophthalmology, Keio University School of Medicine, Tokyo, Japan

[4]Merck & Co., Inc., South San Francisco, California

***Corresponding authors:**

Dr. Lois E.H. Smith, Department of Ophthalmology, Boston Children's Hospital, Boston, MA. 02115; telephone: (+1) 617-919-2529; email: lois.smith@childrens.harvard.edu

Variable	Total N=14	Non-severe ROP N=7	Severe ROP N=7	*P*-value
Mean postnatal days 2-7				
Total energy (kcal/kg/d)	73.6±6.0<br>73 (64 - 85)<br>n=14	74.3±5.7<br>78 (66 - 81)<br>n=7	72.9±6.7<br>72 (64 - 85)<br>n=7	0.68
Enteral energy (kcal/kg/d)	28.5±16.5<br>30 (8 - 57)<br>n=14	39.6±14.0<br>38 (17 - 57)<br>n=7	17.5±10.7<br>13 (8 - 36)<br>n=7	0.024
Parenteral energy (kcal/kg/d)	45.1±15.4<br>43 (21 - 77)<br>n=14	34.8±11.0<br>35 (21 - 52)<br>n=7	55.4±12.0<br>55 (41 - 77)<br>n=7	0.019
Total lipid (g/kg/d)	2.83±0.61<br>2.7 (1.6 - 3.8)<br>n=14	3.14±0.52<br>3.3 (2.5 - 3.8)<br>n=7	2.53±0.56<br>2.5 (1.6 - 3.2)<br>n=7	0.055
Enteral lipid (g/kg/d)	1.50±0.86<br>1.6 (0.4 - 3.0)<br>n=14	2.08±0.71<br>2.0 (0.9 - 3.0)<br>n=7	0.92±0.58<br>0.7 (0.4 - 1.9)<br>n=7	0.019
Parenteral lipid (g/kg/d)	1.33±0.51<br>1.5 (0.4 - 1.9)<br>n=14	1.06±0.56<br>1.2 (0.4 - 1.8)<br>n=7	1.60±0.28<br>1.7 (1.2 - 1.9)<br>n=7	0.10
Mean postnatal days 8-14				
Total energy (kcal/kg/d)	120.5±16.7<br>118 (95 - 149)<br>n=14	130.6±14.2<br>133 (111 - 149)<br>n=7	110.5±12.9<br>112 (95 - 132)<br>n=7	0.017
Enteral energy (kcal/kg/d)	94.6±37.7<br>99 (7 - 149)<br>n=14	121.8±19.2<br>120 (92 - 149)<br>n=7	67.3±31.3<br>67 (7 - 102)<br>n=7	0.002
Parenteral energy (kcal/kg/d)	26.0±24.5<br>16 (0 - 88)<br>n=14	8.8±6.1<br>9 (0 - 18)<br>n=7	43.2±23.9<br>37 (14 - 88)<br>n=7	0.008
Total lipid (g/kg/d)	5.22±0.97<br>5.3 (3.1 - 7.1)<br>n=14	5.60±0.93<br>5.6 (4.0 - 7.1)<br>n=7	4.83±0.91<br>5.0 (3.1 - 5.9)<br>n=7	0.14
Enteral lipid (g/kg/d)	4.43±1.65<br>4.8 (0.4 - 7.1)<br>n=14	5.47±1.02<br>5.5 (3.8 - 7.1)<br>n=7	3.39±1.52<br>3.4 (0.4 - 5.0)<br>n=7	0.019
Parenteral lipid (g/kg/d)	0.78±0.87<br>0.4 (0.0 - 2.7)<br>n=14	0.13±0.14<br>0.2 (0.0 - 0.4)<br>n=7	1.44±0.79<br>1.5 (0.4 - 2.7)<br>n=7	0.0088
Mean postnatal days 15-28				
Total energy (kcal/kg/d)	131.0±18.2<br>135 (103 - 157)<br>n=14	141.7±18.2<br>146 (103 - 157)<br>n=7	120.3±10.9<br>118 (105 - 136)<br>n=7	0.049
Enteral energy (kcal/kg/d)	109.4±43.5<br>117 (17 - 157)<br>n=14	135.0±35.2<br>146 (56 - 157)<br>n=7	83.7±36.3<br>98 (17 - 123)<br>n=7	0.039
Parenteral energy (kcal/kg/d)	21.6±27.0<br>14 (0 - 88)<br>n=14	6.6±17.5<br>0 (0 - 46)<br>n=7	36.6±27.3<br>23 (13 - 88)<br>n=7	0.025
Total lipid (g/kg/d)	5.28±1.28<br>5.5 (3.0 - 7.1)<br>n=14	5.88±1.39<br>6.4 (3.0 - 7.1)<br>n=7	4.68±0.89<br>4.7 (3.4 - 5.9)<br>n=7	0.08
Enteral lipid (g/kg/d)	4.83±1.90<br>5.5 (0.9 - 7.1)<br>n=14	5.74±1.73<br>6.4 (2.0 - 7.1)<br>n=7	3.91±1.70<br>4.3 (0.9 - 5.7)<br>n=7	0.06
Parenteral lipid (g/kg/d)	0.45±0.75<br>0.1 (0.0 - 2.5)<br>n=14	0.14±0.36<br>0.0 (0.0 - 1.0)<br>n=7	0.77±0.93<br>0.2 (0.0 - 2.5)<br>n=7	0.13
Data are presented as mean±standard deviation, median (range) and number of observations.<br>For test between two groups with respect to continuous variables Mann-Whitney U-test was used.				

Supplementary Table 1: Nutritional intake in preterm infants with severe ROP versus no severe ROP.

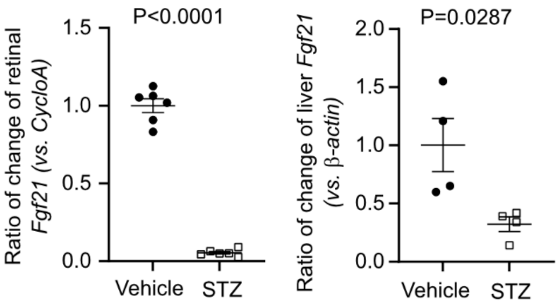

Supplementary Figure 1. Retinal and liver mRNA expression of FGF21 at P10 was decreased in mouse pups with hyperglycemia-induced suppression of physiological vascular development modeling Phase I ROP. mRNA expression of *Fgf21* was compared to the internal control (*CycloA* for retina, *β-actin* for liver) n=6 replicates per group (retina. 6 retinas pooled as n=1), n=4 mice per group (liver). Normality (quantile-quantile plot) and F-test was first conducted and unpaired t-test (liver) or Welch's t-test (retina) was used to compare the groups.

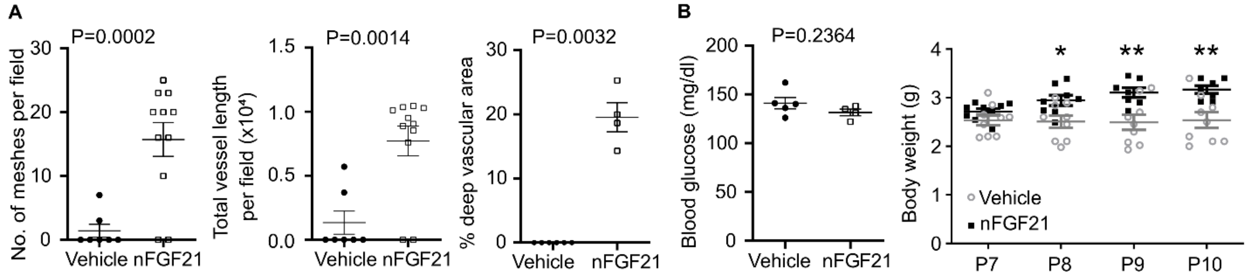

Supplementary Figure 2. (**A**) Native FGF21 (1mg/kg, ip, daily from P7-9) vs. littermate vehicle treatment promoted retinal vessel growth at P10. n=4-11 retinas per group. Normality (quantile-quantile plot) and F-test was first conducted and unpaired t-test or Welch's t-test was used to compare the groups. (**B**) Weight gain in runty pups (2.0 to 3.5 gram at P10). n=7-9 mice per group. Multiple t-test. *P<0.05, **P<0.01.

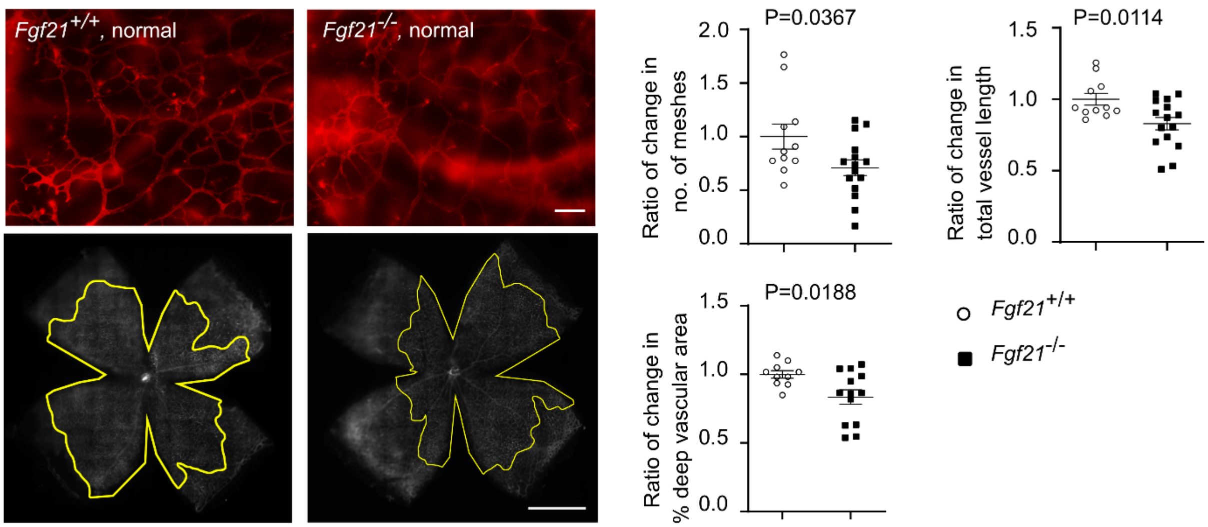

Supplementary Figure 3. FGF21 deficiency delayed physiological retinal vascular development. Decreased retinal vascular network parameters were found in *Fgf21*$^{-/-}$ (knockout) vs. littermate *Fgf21*$^{+/+}$ (WT) mice during normal development at P10. The outlined area (in yellow, bottom) represents the vascular area in the deep plexus. Scale bar: 50μm (top), 1mm (bottom). n=10-15 retinas per group. Normality (quantile-quantile plot) and F-test was first conducted and unpaired t-test was used to compare the groups.

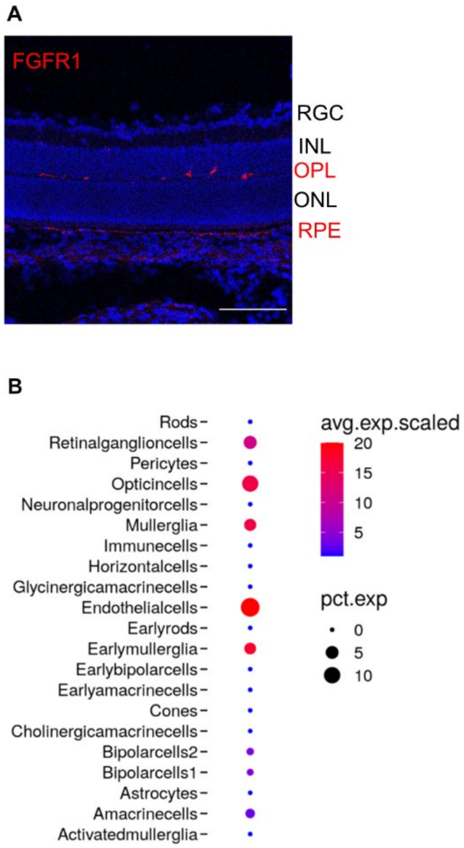

Supplementary Figure 4. Localization of FGFR1 in neonatal mouse retina. (**A**) IHC of FGFR1 (red) on P10 C57BL/6J mouse retina. Blue, DAPI for nucleus. Scale bar, 100µm. (**B**) gene expression of *Fgfr1* at P12 C57BL/6J mouse retinas using Vision Health Research Network Single-Cell Computational platform (https://genap.ca/). Per exp = percent of cells with one or more transcripts for the gene of interest. Avg exp = average transcript count among the cells of a cluster. Transcript counts are scaled around zero (zero = mean across all cells of the dataset).

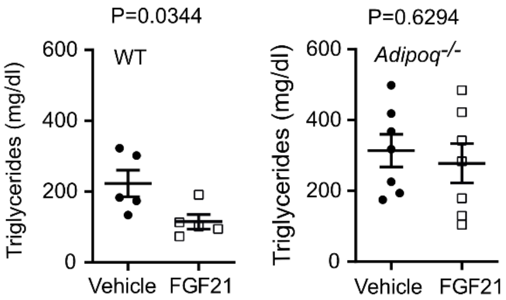

Supplementary Figure 5. FGF21 (PF-05231023) decreased circulating triglyceride in WT mice (n=5 per group), and the impact was abolished in *Adipoq*$^{-/-}$ mice (n=7 mice per group) at P10 in hyperglycemia-associated Phase I ROP model. Normality (quantile-quantile plot) and F-test was first conducted and unpaired t-test was used to compare the groups.
